# Sociodemographic, clinical and treatment characteristics of current rapid-cycling bipolar disorder: a multicenter Chinese study

**DOI:** 10.1186/s40345-024-00332-z

**Published:** 2024-04-09

**Authors:** Jin-jie Xu, Xue-quan Zhu, Shuang Liu, Lu-yu Ding, Bing-bing Fu, Cong-cong Sun, Yan-li Pan, Wei Wang, Ling Zhang

**Affiliations:** 1 Beijing Key Laboratory of Mental Disorders, National Clinical Research Center for Mental Disorders & National Center for Mental Disorders, Beijing Anding Hospital, Capital Medical University, 5 Ankang Lane, Dewai Avenue, Xicheng District, Beijing, 100088 China; 2 Advanced Innovation Center for Human Brain Protection, Capital Medical University, Beijing, 100069 China

**Keywords:** Bipolar disorder, Rapid cycling, Diagnosis duration, Hospitalization, ECT, Treatment response, China, Multicenter study

## Abstract

**Background:**

Rapid cycling bipolar disorder (RCBD), characterized by four or more episodes per year, is a complex subtype of bipolar disorder (BD) with poorly understood characteristics.

**Method:**

This multicenter, observational, longitudinal cohort study enrolled 520 BD patients across seven psychiatric institutions in China from January 2013 to January 2014. Participants were divided into RCBD and non-RCBD (NRCBD) groups based on the frequency of mood episodes in the preceding year. Data collection utilized a standardized form, supplemented by a medical record review, focusing on sociodemographic, clinical, and treatment characteristics. Statistical analysis involved independent samples t-tests, Kruskal–Wallis H tests, Chi-square or Fisher's exact tests, with Bonferroni correction applied to account for multiple comparisons, and multivariable logistic regression to identify characteristics associated with RCBD.

**Results:**

Among the BD cohort, 9.4% were identified as current RCBD. Compared to NRCBD, RCBD patients had a shorter duration from the first psychiatric consultation to the diagnosis of BD, a reduced duration of their longest period of euthymia, a lower proportion of lifetime hospitalization history due to BD, and less use of electroconvulsive therapy (ECT) within the last 12 months. Additionally, they presented higher baseline scores on the Mood Disorder Questionnaire (MDQ) and the Brief 16-item Quick Inventory of Depressive Symptomatology Self-Report (QIDS-SR16). However, after applying the Bonferroni correction, these differences were not statistically significant. Multivariable logistic regression analysis identified three factors that were independently associated with RCBD: time from first psychiatric consultation to BD diagnosis (Odds Ratio [OR] = 0.512, *P* = 0.0416), lifetime hospitalization history due to BD (OR = 0.516, *P* = 0.0476), and ECT treatment within the past 12 months (OR = 0.293, *P* = 0.0472).

**Conclusion:**

This study revealed that the duration from first psychiatric consultation to BD diagnosis, lifetime hospitalization history due to BD, and ECT treatment in the past year were associated with RCBD. Recognizing these factors could contribute to enhance the early identification and clinical outcomes of RCBD.

*Trial Registration Number* Registry ClinicalTrials.gov NCT01770704. Date of Registration: First posted on January 18, 2013.

## Introduction

Bipolar disorder (BD) is a prevalent mood disorder marked by emotional swings, affecting about 2.4% of adults globally, disrupting daily life and contributing to global health loss (American Psychiatric Association et al. 2013; Merikangas et al. 2011; Zhang et al. 2017a; Nierenberg et al. 2023). Rapid Cycling BD (RCBD), a subset of BD identified by Dunner and Fieve in 1974, involves experiencing four or more mood episodes within a year (Dunner and Fieve 1974). Despite debates regarding its permanence, with some researchers suggesting RCBD may be a transient rather than a distinct subtype of BD (Carvalho et al. 2014), the Diagnostic and Statistical Manual of Mental Disorders, Fifth Edition (DSM-5) recognizes it as a longitudinal course specifier. While affecting 22.3–35.5% of BD patients and raising important clinical and therapeutic concerns, RCBD's underlying mechanisms remain largely unexplored, emphasizing the need for further research (Miola et al. 2023a).

Recent studies highlight distinct features of RCBD compared to non-RCBD (NRCBD). RCBD patients predominantly experience frequent depressive episodes and atypical features and are often diagnosed with bipolar II disorder (BD-II) (Miola et al. 2023a; Kupka et al. 2003). This subtype is characterized by an earlier onset, a prolonged course of illness, and increased psychiatric comorbidities (Carvalho et al. 2014; Miola et al. 2023a; Valentí et al. 2015; Kato et al. 2020; Takano et al. 2023; Antonietta Furio et al. 2021; Yao et al. 2023; Prisciandaro et al. 2019), often coupled with increased substance and alcohol abuse(Carvalho et al. 2014; Gordon-Smith et al. 2020), and adverse childhood experiences(Miola et al. 2023a, 2023b; Yao et al. 2023). Clinically, RCBD presents with greater disease severity, increased suicide attempts(Carvalho et al. 2014; Miola et al. 2023a, 2023b; Valentí et al. 2015; Takano et al. 2023), and a higher risk of recurrence and hospitalization(Kato et al. 2020; Miola et al. 2023b; Sengupta and Jena 2022). In terms of treatment, RCBD is often resistant to conventional pharmacotherapy, necessitating a more complex treatment regimen, often involving polypharmacy. However, the link between RCBD and the use of antidepressants remains contentious(Ghaemi et al. 2010; El-Mallakh et al. 2015; Strawbridge et al. 2022; Schneck et al. 2008), and research on the efficacy of electroconvulsive therapy (ECT) in treating RCBD is scarce(Ninke and Groene 2023; Minnai et al. 2011; Huber and Burke 2015). In summary, an optimal treatment strategy for RCBD has yet to be established (Strawbridge et al. 2022).

Despite extensive research, many clinical and treatment characteristics associated with RCBD remain unclear or controversial (Strawbridge et al. 2022; Thase 2013; Amsterdam et al. 2017; Roosen and Sienaert 2022; Tao et al. 2023; Munkholm 2022), especially with limited studies from China. This study represents a comprehensive multicenter investigation across the nation, aiming to: (1) investigate the current prevalence of RCBD in a Chinese sample; and (2) compare the sociodemographic, clinical, and treatment characteristics of RCBD with its non-RCBD counterpart.

## Methods

### Study design

The data for this analysis were obtained from the “Clinical Management of Bipolar Disorder in China,” a comprehensive, multicenter, observational, and longitudinal cohort study (Registration number: NCT01770704) initiated by the Chinese Society of Psychiatry in 2013 (Zhang et al. 2016, 2017b). This investigation was divided into a retrospective stage, commencing one year prior to patient recruitment and concluding upon informed consent acquisition, and a prospective stage, starting with informed consent and ending after a nine-month follow-up period post-enrollment. To reflect a representative snapshot of the clinical handling of BD across China, the study engaged seven representative psychiatric institutions, including large specialty hospitals and general hospitals with psychiatric outpatient services, as the pivotal research sites. These included the Beijing Anding Hospital, the Peking University Institute of Mental Health, the Shanghai Mental Health Center, the Second Affiliated Hospital of Zhejiang University School of Medicine, the Shenzhen Mental Health Center, the Xijing Hospital, and the First Affiliated Hospital of Kunming Medical University. These centers enrolled a cohort of 50 to 150 patients each, resulting in an aggregate of 520 patients across the enrollment window from January 2013 to January 2014.

### Study population

Inclusion criteria were as follows: adults age ≥ 18 years, male or female, with a diagnosis of bipolar I disorder (BD-I) or BD-II, and having experienced a minimum of one mood episode in the twelve to three months preceding the study's onset. As described in prior literature, the diagnosis of BD-I and BD-II was based on the Diagnostic and Statistical Manual of Mental Disorders, Fourth Edition (DSM-IV) criteria, as confirmed by two consultant psychiatrists with over 15 years clinical experience(Zhang et al. 2017b). Patients who were unable to comprehend the interview content were excluded. Participants were stratified into two cohorts: the current RCBD group, characterized by four or more mood episodes in the year preceding enrollment, and the NRCBD group, with fewer than four episodes.

### Data collection

Our study utilized a standardized data collection form, complemented by a medical record review to ensure the completeness and accuracy of the information. Sociodemographic characteristics, clinical characteristics, and treatments previously associated or potentially associated with RCBD were included in this analysis. Sociodemographic characteristics included gender, age, education, occupational status, and residential status. Lifetime clinical characteristics included substance abuse, family history of mental disorders, the type of BD, age at onset, age at diagnosis, duration from the first psychiatric consultation to the diagnosis of BD, other psychiatric diagnoses prior to BD, polarity of the initial episode, psychotic feature, longest duration of euthymia post-diagnosis, hospitalizations history, and lifetime suicide attempts. Clinical characteristics of the past 12 months included the number of episodes of each polarity (manic, hypomanic, depressive, and mixed), hospitalization history, and suicide attempts. Treatment data for the past 12 months included the number and categories of psychotropic medications (including mood stabilizers, antipsychotics, antidepressants, and benzodiazepines) and the administration of ECT.

Baseline severity of depressive symptoms was assessed using the Brief 16-item Quick Inventory of Depressive Symptomatology Self-Report (QIDS-SR16), a scale designed to quantify the severity of depressive symptomatology across 16 items, covering the nine symptomatic domains as defined by the DSM-IV criteria for depressive episodes(Feng et al. 2016). Meanwhile, the Mood Disorder Questionnaire (MDQ) served as a screening instrument to detect a history of manic or hypomanic symptoms. The MDQ's design facilitates the retrospective assessment of manic or hypomanic symptom history, the evaluation of concurrent symptomatology, and the assessment of related functional impairments (Yang et al. 2011). This study's referential period for these assessments was demarcated as the week preceding the evaluation.

### Statistical analysis

SAS statistical software (Version 9.4, SAS Institute Inc., USA) was used for all data processing and analysis. Descriptive statistics for continuous variables conforming to a normal distribution were articulated as mean ± standard deviation (SD), and the independent samples t-test was employed for intergroup comparisons. Non-normally distributed continuous variables were depicted using medians and interquartile ranges [M (P25, P75)], with the Kruskal–Wallis H test applied to discern differences between groups. Categorical variables were presented in frequencies or percentages (%) and compared across cohorts using the Chi-square (χ2) test or Fisher's exact test, where appropriate. We adjusted for multiple comparisons using Bonferroni correction (0.05/41) requires p < 0.0012). A logistic regression model was used to analyze the potential factors associated with RCBD. In this model, the presence of RCBD was set as the dependent variable, and age, gender, duration from first psychiatric consultation to diagnosis of BD, lifetime hospitalization history due to BD, lifetime suicide attempts, and ECT treatment in the last 12 months were included as independent variables. All tests were two-sided and statistical significance was set at* P* < 0.05.

## Results

### Sociodemographic and clinical characteristics between RCBD and NRCBD

This study included 520 patients with BD, averaging 35.65 years, of whom 48.46% were male. Among this cohort, 49 individuals (9.4%) were identified as current RCBD. No significant differences were observed in sociodemographic characteristics such as gender, age, education level, occupational status, and residential status between the two groups (*P* > 0.05). Lifetime clinical characteristics revealed that the RCBD group had a shorter duration from the first psychiatric consultation to the diagnosis of BD (mean = 2.46 years vs. 3.57 years; *P* = 0.0243), and notably, the longest period of euthymia post-diagnosis was significantly shorter in the RCBD group compared to the NRCBD group (mean = 573.85 days vs. 918.67 days; *P* = 0.0180). Furthermore, the RCBD group exhibited a lower proportion of lifetime hospitalization history due to BD (65.31% vs. 81.32%; *P* = 0.0079). However, none of these outcomes met the stringent criteria required by the Bonferroni correction for statistical significance. No significant differences were found in other lifetime clinical characteristics (*P* > 0.05). Detailed data are presented in Table 1.

**Table 1:** Sociodemographic and clinical characteristics between RCBD and NRCBD. Significance with Bonferroni correction requires p < 0.0012 (0.05/41)

Variables	Total (n = 520)	RCBD (n = 49)	NRCBD (n = 471)	*t/Z/χ2*	*P*
Sociodemographic characteristics					
Gender (male)	252(48.46)	20(40.82)	232(49.26)	1.2660	0.2605
Age (years)	35.65 ± 13.23	34.29 ± 13.00	35.80 ± 13.26	− 0.76	0.4499
Education (years)	13.10 ± 3.40	13.18 ± 3.52	13.09 ± 3.39	0.19	0.8519
Occupational status				0.4617	0.7939
Permanent employment	221(42.50)	22(44.90)	199(42.25)		
Temporary employment	69(13.27)	5(10.20)	64(13.59)		
Unemployed	230(44.23)	22(44.90)	208(44.16)		
Residential status				–	0.5790
Living alone	42(8.08)	5(10.20)	37(7.86)		
Cohabitation	478(91.92)	44(89.80)	434(92.14)		
Lifetime clinical characteristics					
Substance abuse	36(6.92)	2(4.08)	34(7.22)	–	0.5620
Family history of mental disorders	151(29.04)	16(32.65)	135(28.66)	0.3430	0.5581
Bipolar type				0.7280	0.3935
Type I	399(76.73)	40(81.63)	359(76.22)		
Type II	121(23.27)	9(18.37)	112(23.78)		
Age at onset (years)	30.39 ± 12.21	31.01 ± 13.11	30.32 ± 12.12	0.38	0.7059
Age at diagnosis (years)	32.28 ± 12.46	31.61 ± 13.0	32.35 ± 12.41	− 0.40	0.6930
Time from first psychiatric consultation to diagnosis of BD (years) (Mean ± SD)	3.46 ± 6.13	2.46 ± 6.81	3.57 ± 6.05	5.0762	**0.0243**
Psychiatric diagnosis prior BD				4.4707	0.2149
Major depressive disorder	227(43.65)	24(48.98)	203(43.10)		
Schizophrenia	84(16.15)	3(6.12)	81(17.20)		
Anxiety Disorder	39(7.50)	3(6.12)	36(7.64)		
Other mental disorders	170(32.69)	19(38.78)	151(32.06)		
Polarity of initial episode				–	0.0775
Manic episode	172(33.08)	11(33.08)	161(22.45)		
Hypomanic episode	19(3.65)	2(4.08)	17(3.61)		
Depressive episode	307(59.04)	31(63.27)	276(58.60)		
Mixed episode	22(4.23)	5(10.20)	17(3.61)		
Psychotic feature	217(41.73)	18(36.73)	199(42.25)	0.5553	0.4562
Longest duration of euthymia post-diagnosis (days) (n = 247)	882.38 ± 1318.71	573.85 ± 841.84	918.67 ± 1360.67	5.6006	**0.0180**
Lifetime hospitalization history due to BD	415(79.81)	32(65.31)	383(81.32)	7.0596	**0.0079**
Lifetime number of hospitalizations due to BD	1.92 ± 1.51	1.91 ± 1.55	1.92 ± 1.51	0.4041	0.5250
Lifetime suicide attempts	54(10.38)	8(16.33)	46(9.77)	2.0524	0.1520
Number of lifetime suicide attempts	0.17 ± 0.67	0.29 ± 0.84	0.15 ± 0.64	0.7547	0.3850
Clinical characteristics in the past 12 months					
Number of episodes in the last 12 months (Mean ± SD)					
Manic episode	0.30 ± 0.75	1.22 ± 1.79	0.20 ± 0.43	21.4834	< 0.001
Hypomanic episode	0.73 ± 1.04	1.73 ± 2.44	0.63 ± 0.69	14.98	< 0.001
Depressive episode	1.03 ± 1.07	2.63 ± 2.21	0.86 ± 0.69	71.65	< 0.001
Mixed episode	0.15 ± 0.61	0.63 ± 1.65	0.10 ± 0.31	17.09	< 0.001
Total number of episodes in the last 12 months (Mean ± SD)	2.22 ± 1.91	6.24 ± 4.01	1.80 ± 0.71	150.7186	< 0.001
Hospitalization history due to BD in the past 12 months	348(66.92)	27(55.10)	321(68.51)	3.4150	0.0646
Number of hospitalizations due to BD in the past 12 months	1.23 ± 0.53	0.97 ± 0.59	1.04 ± 0.67	0.4083	0.5228
Suicide attempts in the past 12 months	32(6.15)	5(10.20)	27(5.73)	-	0.2107
Number of suicide attempts in the past 12 months	0.08 ± 0.35	0.18 ± 0.75	0.06 ± 0.27	0.2603	0.6099

*BD* bipolar disorder; *RCBD* rapid cycling bipolar disorder; *NRCBD* non-rapid cycling bipolar disorder

### Treatments in the last 12 months and baseline status between RCBD and NRCBD

Over the past 12 months, 78.08% of patients were prescribed a regimen of three or more psychotropic drugs. Mood stabilizers, antipsychotics, antidepressants, and benzodiazepines were used in 91.5%, 84.62%, 39.62%, and 17.88% of the cases, respectively, with no statistical difference between the RCBD and NRCBD groups regarding the use of these medication categories (*P* > 0.05). Specifically, mood stabilizers were administered as follows: lithium (51.9%), valproic acid (61.4%), carbamazepine (0.2%), and lamotrigine (8.5%), again showing no significant statistical difference between the groups (*P* > 0.05). Usage patterns of mood stabilizers indicated that 62.5% of patients were on one, 27.7% on two, and 1.4% on three different mood stabilizers, with no significant intergroup variance (*P* > 0.05). However, the incidence of ECT during hospitalization in the preceding 12 months was notably lower in the RCBD group compared to the NRCBD group (11.1% vs. 29.6%; *P* = 0.0403). In addition, the RCBD group had a higher baseline score on the first section of the MDQ (mean = 3.92 vs. 3.04; *P* = 0.0105) and on the QIDS-SR16 (mean = 9.10 vs. 7.60; *P* = 0.0230) than the NRCBD group. However, none of these observations successfully surpassed the stringent significance threshold set by the Bonferroni correction. Detailed data are presented in Table 2.

**Table 2:** Treatments in the last 12 months and baseline status between RCBD and NRCBD. Significance with Bonferroni correction requires p < 0.0012 (0.05/41)

Variables	Total (n = 520)	RCBD (n = 49)	NRCBD n = 471)	t/Z/χ2	*P*
Number of psychotropic medications				-	0.7004
No medication	10(1.92)	1(2.04)	2(1.91)		
One medication	15(2.88)	1(2.04)	14(2.97)		
Two medications	89(17.12)	11(22.45)	78(16.56)		
Three and more medications	406(78.08)	36(73.47)	370(78.56)		
Mood stabilizers	476(91.5)	46(93.9)	430(91.3)	-	0.7869
Lithium	270(51.9)	24(49.0)	246(52.2)	0.1878	0.6648
Valproic acid	319(61.4)	32(65.3)	287(60.9)	0.3578	0.5498
Carbamazepine	1(0.2)	0(0.0)	1(0.2)	-	1.0000
Lamotrigine	44(8.5)	8(16.3)	36(7.6)	-	0.0539
Number of mood stabilizers				-	0.3246
One mood stabilizer	325(62.5)	30(61.2)	295(62.6)		
Two mood stabilizers	144(27.7)	14(28.6)	130(27.6)		
Three mood stabilizers	7(1.4)	2(4.1)	5(1.1)		
Antipsychotics	440(84.62)	41(83.67)	399(84.71)	0.0369	0.8477
Antidepressants	206(39.62)	22(44.90)	184(39.07)	0.6311	0.4270
Benzodiazepines	93(17.88)	10(20.41)	83(17.62)	0.2346	0.6281
ECT during hospitalization in the last 12 months (n = 348)	98(28.2)	3(11.1)	95(29.6)	4.2060	**0.0403**
MDQ Part 1 scores (Mean ± SE)	3.13 ± 3.19	3.92 ± 2.93	3.04 ± 3.21	5.5477	**0.0105**
QIDS-SR16 score (Mean ± SE)	7.75 ± 5.47	9.10 ± 5.05	7.60 ± 5.49	5.1690	**0.0230**

bolded to represent p values less than 0.05.*BD* bipolar disorder; *RCBD* rapid cycling bipolar disorder; *NRCBD* non-rapid cycling bipolar disorder; *ECT* electroconvulsive therapy; *MDQ* Mood Disorder Questionnaire; *QIDS-SR16* Brief 16-item Quick Inventory of Depressive Symptomatology Self-Report

### Multivariable modeling of factors associated with RCBD

The study employed a logistic regression model to explore potential factors associated with RCBD. The analysis indicated significant inverse correlations with RCBD for several variables: the duration from first psychiatric consultation to diagnosis of BD (odds ratio [OR] = 0.512, *P* = 0.0416), lifetime hospitalization history due to BD (OR = 0.516, *P* = 0.0476), and ECT treatment in the last 12 months (OR = 0.293, *P* = 0.0472). Detailed data are presented in Table 3.

**Table 3:** Multivariable modeling of factors associated with RCBD

Variables	β	SE	Wald χ2	*P*	OR	95%CI
Age	− 0.01	0.01	0.3508	0.5537	0.993	0.970,1.017
Gender	− 0.14	0.16	0.7495	0.3866	0.762	0.412,1.409
Time from first psychiatric consultation to diagnosis of BD (years) (reference < 1)	− 0.33	0.16	4.1534	**0.0416**	0.512	0.269,0.975
Lifetime hospitalization history due to BD	− 0.33	0.17	3.9254	**0.0476**	0.516	0.268,0.993
Lifetime suicide attempts	0.34	0.22	2.4952	0.1142	1.991	0.847,4.680
Received ECT in the last 12 months	− 0.61	0.31	3.9367	**0.0472**	0.293	0.087,0.985

bolded to represent p values less than 0.05.*BD* bipolar disorder; *RCBD* rapid cycling bipolar disorder; *ECT* electroconvulsive therapy

## Discussion

In this study, 9.4% of BD patients exhibited RC characteristics within the recent 12 months. This finding aligns with a retrospective and prospective follow-up of 1,261 BD patients conducted by Miola A et al., where 9.36% of BD patients reported RC characteristics over the past 12 months (Miola et al. 2023b). However, Miola A et al.’s mixed method systematic meta-review reported one-year prevalence of 22.3% and a lifetime prevalence of 35.5% (Miola et al. 2023a). Such discrepancies might be attributed to genetic, regional, cultural, and therapeutic differences.

In our study, the average diagnostic duration for RCBD was 2.46 years, shorter than the NRCBD (3.57 years), indicating earlier diagnosis for RCBD patients. This might be attributed to the more frequent mood fluctuations in RCBD prompting earlier medical intervention, providing clinicians with more apparent clinical cues.

In this study, we noted that the lifetime hospitalization rate due to BD for RCBD patients was lower than their NRCBD counterparts (65.31% vs. 81.32%). These findings align with Gigante et al.’s research (Gigante et al. 2016) within the Brazilian Bipolar Disorder Research Network. Despite both groups having comparable average lifetime hospitalizations (RCBD = 3.6 ± 6.6 times; NRCBD = 3.4 ± 7.9 times), a significantly higher proportion of NRCBD patients were hospitalized compared to the RCBD group (71.8% vs. 60.4%). Several factors might account for the lower lifetime hospitalization rates observed in RCBD patients. Primarily, earlier identification and intervention for RCBD symptoms could have facilitated timely and effective treatments, preventing further symptom exacerbation. Additionally, instead of prolonged inpatient care, RCBD patients might prefer outpatient or day-care treatments. Contradictory findings by Buoli et al. reported a higher annual hospitalization rate for RCBD patients compared to NRCBD patients (53.5% vs. 39.9%) (Buoli et al. 2019). Moreover, research outcomes by Miola A et al. highlighted a 3.21-fold increase in the average prospective relapse rate for RCBD patients over a year-long follow-up [2.44 (1.83–3.06) times/year], compared to 0.76 (0.70–0.82) times/year for the NRCBD group (Miola et al. 2023b). Therefore, although some studies suggest lower hospitalization rates for RCBD patients, their higher relapse frequency underscores potential challenges in their long-term therapeutic management.

In this study, we observed parallel pharmacological treatment patterns in RCBD and NRCBD patients, including comparable utilization of mood stabilizers, antipsychotics, and antidepressants without significant differences. The optimal treatment strategy for RCBD remains elusive due to the limited and diverse nature of the evidence (Strawbridge et al. 2022). The National Institute for Health and Care Excellence (NICE) guidelines recommend a similar treatment approach for both RCBD and NRCBD patients, advocating for the use of second-generation antipsychotics for mania and advising against the continuation of antidepressants (Centre and for Mental H. 2014). Research on the role of antidepressants in RCBD yields mixed results. The STEP-BD studies (Ghaemi et al. 2010; El-Mallakh et al. 2015; Schneck et al. 2008) indicate that prolonged use of antidepressants might exacerbate RC symptoms and elevate the risk of depressive episodes. In contrast, Amsterdam JD’s(Amsterdam et al. 2013) randomized, double-blind, placebo-controlled study comparing fluoxetine with lithium monotherapy in patients with rapid and non-rapid-cycling BD-II reported no significant differences in depressive relapse or treatment-emergent mood conversion between the groups. Similarly, Strawbridge's meta-analysis (Strawbridge et al. 2022) underscores the potential benefits of specific SSRIs and bupropion. Given these study's findings, further research is imperative to explore the long-term outcomes and real-world effectiveness of these treatments in a larger, more diverse cohort.

Our study reported for the first time that RCBD patients showed a markedly lower frequency of undergoing ECT in the past 12 months compared to their NRCBD counterparts. ECT's efficacy is established in treating acute episodes of BD, particularly in medication-resistant cases with severe symptoms or self-harm risk. Notably, RCBD and ultra-rapid cycling BD patients might exhibit enhanced ECT responsiveness. However, this evidence stems mainly from case studies or small-scale research. For instance, an open-label study conducted by Mosolov et al. in 2021 on rapid and ultra-rapid cycling BD patients demonstrated a significant reduction in mood episodes following an acute ECT course (Mosolov et al. 2021). Additionally, a 2011 study by Minnai et al. indicated that maintenance ECT substantially reduced the number of morbid days per annum in RCBD patients, highlighting its long-term prophylactic effects (Minnai et al. 2011). Their multivariate analysis identified young age, male gender, BD-II, and hyperthymic temperament as predictors of a favorable maintenance ECT response. Hence, despite limited evidence, acute ECT is advocated for treating medication-resistant episodes in RCBD, with maintenance ECT as a viable option for those showing initial improvement or experiencing pharmacotherapy relapse.

In this study, we observed that RCBD patients exhibited significantly higher scores on the MDQ and QIDS-SR16, implying suboptimal disease control and therapeutic efficacy in the RCBD group. It was reported in previous studies that RCBD patients tend to have lower responses to both pharmacological and psychotherapeutic interventions. For instance, a meta-analysis by Hui et al. involving nine studies involving a total of 1442 BD patients revealed a notably reduced response to lithium treatment in patients with RCBD symptoms compared to those without (Hui et al. 2019). Moreover, another meta-analysis including 30 randomized trials and 2,266 patients evaluated 16 independent pharmacological interventions and one psychotherapeutic approach for RCBD, and found that despite the large number of potential treatment modalities available, an optimal therapeutic regimen for RCBD remains elusive (Strawbridge et al. 2022).

## Limitations

The present study has several limitations. Firstly, this research employs an observational design, thereby precluding the establishment of causal relationships. Secondly, the study only encompasses the episodes occurring within the past year, and thus may not fully capture the participants' earlier patterns of rapid cycling. This limitation could have implications for the epidemiological and clinical interpretation of the study. Thirdly, the MDQ and QIDS-SR16 scores are based on patient self-reporting, which may introduce potential recall bias or reporting prejudices. It is important to note that the MDQ serves as a screening tool for manic or hypomanic symptoms, without assessing symptom severity. Lastly, this study primarily relies on baseline data and does not offer insights into the long-term prognosis, limiting a comprehensive understanding of the disease trajectory in RCBD patients. Future research should incorporate prospective longitudinal studies to elucidate the long-term course of RCBD, including a comprehensive assessment of lifetime mood episodes. Additionally, employing more precise diagnostic tools, such as the Altmann Mania Rating Scale or the Young Mania Rating Scale, is crucial to quantify the severity of manic or hypomanic symptoms.

## Conclusion

In conclusion, our findings indicate that 9.4% of BD patients exhibit RCBD, which is associated with shorter diagnosis duration, lower lifetime hospitalization rate due to BD, and lower rates of ECT treatment within the past year. However, RCBD patients also showed poorer treatment outcomes. These results emphasize the need for better treatment strategies for RCBD patients in China. It is crucial to improve the training and implementation of standardized treatments to enhance therapeutic outcomes for this group of patients.

## Data Availability

The data that support the fndings of this study are not openly available due to reasons of sensitivity and are available from the corresponding author upon reasonable request. Data are located in controlled access data storage at Beijing Anding Hospital.

## Abbreviations

BD: Bipolar Disorder
RCBD: Rapid Cycling Bipolar Disorder
NRCBD: Non-Rapid Cycling Bipolar Disorder
DSM-5: Diagnostic and Statistical Manual of Mental Disorders, Fifth Edition
DSM-IV: Diagnostic and Statistical Manual of Mental Disorders, Fourth Edition
ECT: Electroconvulsive Therapy
MDQ: Mood Disorder Questionnaire
QIDS-SR16: Quick Inventory of Depressive Symptomatology Self-Report (16-item)
SAS: Statistical Analysis System
OR: Odds Ratio
BD-II: Bipolar Disorder Type II
STEP-BD: Systematic Treatment Enhancement Program for Bipolar Disorder
NICE: National Institute for Health and Care Excellence
SSRIs: Selective Serotonin Reuptake Inhibitors
